# Assessing vaccine hesitancy among healthcare providers in Brazil: the influence of vaccine status and professional experience

**DOI:** 10.1016/j.jped.2024.09.001

**Published:** 2024-10-05

**Authors:** Luiz Gustavo Almeida, Renato de Ávila Kfouri, Natalia Pasternak Taschner, Eduardo Jorge da Fonseca Lima, Ronaldo Pilati

**Affiliations:** a Instituto Questão de Ciência, São Paulo, SP, Brazil; b Sociedade Brasileira de Imunizações, São Paulo, SP, Brazil; c Center of Science and Society, Columbia University, New York, USA; d Instituto de Medicina Integral Professor Fernando Figueira, Recife, PE, Brasil; e Departamento de Psicologia Social e do Trabalho, Universidade de Brasília, Brasilia, DF, Brazil

**Keywords:** Vaccine hesitancy, Pediatricians, COVID-19 vaccines, Medical residency, Immunization

## Abstract

**Objective:**

Assess the occurrence of vaccine hesitancy among pediatricians and their patients and identify potential predictors to mitigate hesitancy among them.

**Methods:**

The study is a cross-sectional survey using an online questionnaire sent to pediatricians affiliated with the Brazilian Society of Pediatrics. The data was analyzed using statistical methods such as exploratory factor analysis, principal component analysis, correspondence analysis, and generalized linear mixed models.

**Results:**

A total of 982 respondents, with a majority being females (77.4%), participated in the research. Among them, the proportion of pediatricians with complete vaccine status was 41.14%, while 90.6% had undergone medical residency. Furthermore, 9.3% worked in public healthcare settings, 30.4% in private settings, and 60.3% in mixed healthcare settings. The analysis revealed a significant association between vaccine status and pediatricians’ misconceptions about COVID-19 vaccines, with those having complete vaccine status showing lower misconceptions (mean difference of -0.15, p = 0.010). Moreover, pediatricians with medical residency experience exhibited fewer misconceptions about COVID-19 vaccines (mean difference of -0.33, p = 0.002). Additionally, correspondence analysis unveiled the presence of two distinct profiles among pediatricians, showcasing variations in vaccine education, professional experience, and vaccine confidence perceptions.

**Conclusion:**

The study highlights the influence of vaccine status and medical residency experience on pediatricians’ attitudes and misconceptions about vaccines, emphasizing the need for targeted educational interventions to promote vaccine confidence and combat hesitancy within the healthcare provider community.

## Introduction

In Brazil, the existence of a comprehensive vaccination program run by the National Immunization Program (PNI) is of paramount importance for pediatric healthcare. This program provides a thorough vaccination schedule that safeguards children from infectious diseases across the country. The vaccination calendar encompasses vaccines against a wide array of illnesses, including hepatitis B, tuberculosis, polio, diphtheria, tetanus, whooping cough, rotavirus, measles, mumps, pneumococcal and meningococcal disease, rubella, varicella, Haemophilus influenzae b, Yellow fever, hepatitis A, HPV, and notably, COVID-19.^1^

Pediatricians and healthcare providers across Brazil hold a pivotal role in the successful execution of this vaccination schedule. According to Scheffer M, there are 48,654 pediatricians in Brazil, which represents 9.8% of all registered medical specialists. Of these, 75.6% are women. These healthcare professionals are the primary and most dependable source of information for patients.^2^

However, the emergence of vaccine hesitancy, characterized by the delay or refusal of vaccines despite their availability, has raised significant concerns. This hesitancy poses challenges for both the PNI and pediatric vaccination efforts.^3^

Vaccine hesitancy heightens the risk of disease outbreaks, and re-emerging diseases, and weakens the PNI. Additionally, healthcare providers may encounter difficulties when attempting to convince hesitant parents to adhere to the recommended vaccination schedule.^4^

Furthermore, vaccine hesitancy can have a profound impact on the practices and perceptions of pediatricians. These healthcare providers are instrumental in advocating for immunization, yet countering misinformation and misconceptions when faced with vaccine-hesitant parents can be a source of frustration and concern. Pediatricians often find themselves in the challenging position of dispelling myths and addressing concerns, further complicating the immunization process.^5^, ^6^, ^7^, ^8^

The primary goal of this study was to analyze the occurrence of vaccine hesitancy among pediatricians affiliated with the Brazilian Society of Pediatrics and their patients. Additionally, the study aimed to assess predictors that could potentially help mitigate vaccine hesitancy among pediatricians.

## Methods

The study is descriptive, cross-sectional, and conducted through an online self-administered questionnaire via Google Forms. Invitations were sent to members of the Brazilian Society of Pediatrics, representing 37.31% of Brazilian pediatricians, from 24/02/2023 to 20/04/2023, with monthly reinforcements through messaging apps. Participants provided informed consent, ensuring voluntary participation, confidentiality, anonymity, and their right to withdraw at any time without any consequences. The study addressed vaccine hesitancy among healthcare providers with sensitivity to their professional integrity and beliefs. It was approved by the ethics committee of the Instituto de Medicina Integral Prof. Fernando Figueira (approval process number: 5.920.518).

### Survey instrument

A multidisciplinary team, comprising pediatricians, science communicators, and psychologists, collaborated to develop the survey instrument. The survey encompassed questions to comprehensively capture insights from participants and it comprised multiple-choice questions, open-ended responses, and Likert scale questions. The sections covered a spectrum of topics related to vaccine hesitancy and pediatrician attitudes towards vaccinations. The instrument included five sections: **Section 1:** Hesitation in Professional Practice. It has eight items evaluating the hesitation faced by professionals in their practice; **Section 2:** Handling Hesitant Families. Five Likert scale questions were used to comprehend participants’ strategies when addressing families with vaccine hesitancy; **Section 3:** Overcoming Challenges with Hesitant Families. Six Likert scale items and one multiple-choice item investigating challenges participants encountered in addressing vaccine hesitancy; **Section 4:** Pediatrician Vaccine Hesitancy and Misconceptions. Participants’ viewpoints on vaccines were assessed using a Likert scale with 11 statements. Among these, five statements were extracted from the Vaccine Hesitancy 5C scale, designed to evaluate dimensions like confidence, constraints, collective responsibility, complacency, and calculation concerning vaccines.^10^ The additional six statements explored beliefs such as vaccine misconceptions and concerns about DNA modification due to COVID-19 vaccines; **Section 5:** General Information About Participants in which the authors asked for demographic information such as gender, academic background, workplace setting (public and/or private) and age.

The survey was pilot-tested with a selected group of pediatricians to ensure the clarity of questions, ease of navigation, and overall usability.

### Statistical analysis

The analysis was conducted using Jamovi version 2.4.5 and R version 4.3.1.

#### Exploratory factor analysis (EFA)

EFA identifies patterns, organizes similar questions, reveals underlying structures, and simplifies complex data. For the 5Cs-related items, the authors set the extraction of one factor through Principal Axis extraction with oblimin rotation. Other Pediatrician Perspectives on Vaccines items employed maximum likelihood extraction due to response variability, with oblimin rotation for correlations. Parallel analysis determined the number of factors to extract. Model Fit Measures were RMSEA and TLI; the chi-square test checked the model. RMSEA below 0.05 and TLI above 0.9 were suitable. Bartlett's Test (*p <* 0.05) and KMO Measure (above 0.8 excellent, 0.61–0.79 good) verified assumptions. McDonald's ω reported Scale Reliability (0.61–0.79 good).

#### Principal component analysis (PCA)

The scale of beliefs in misinformation about pediatric COVID-19 vaccines was developed using the PCA method based on the clustering of questions identified by EFA. PCA is a statistical technique that simplifies complex data by uncovering underlying patterns in related variables. In the present study, according to the best factorial structure found through exploratory factor analysis, the authors employed Principal Component Analysis to generate scores for each pediatrician. These scores were then used as the dependent variable in the linear regression model. This process involved calculating principal components that combine multiple questions and explain most of the variation in the data.^9^ This allowed us to create a condensed scale of beliefs in vaccine misinformation while retaining the essential insights obtained through EFA, facilitating result analysis.

#### Multiple correspondence analysis

To assess group differences, the authors employed Multiple Correspondence Analysis. This technique explores relationships among categorical variables. In this study, the authors applied this method to variables including pediatricians’ vaccine status, vaccine confidence, comfort in educating families about vaccines, medical residency, possession of Master's or Doctorate degrees, and workplace settings.

#### Generalized linear models (GLM)

The authors used a Generalized Linear Model (GLM) analysis with a Gamma distribution and Identity link function to examine how the COVID-19 Vaccines Misconception Scale (the dependent variable) relates to vaccination status, gender, and medical residency experience (the three independent variables).

## Results

### Sample demographics

The survey comprised 982 participants recruited via email and Whatsapp, with a predominantly female representation (77.4%) and a small portion (0.1%) identified as non-binary. Most participants had medical residency experience (90.6%) and incomplete vaccine status (58.9%). Participants came from diverse educational backgrounds, with 24% holding a master's degree and 10.8% possessing a doctoral degree. They hailed from various regions across Brazil, with the highest numbers originating from the Southeast (21%) and Northeast (14.8%), trailed by the South (7.8%) and Central-West (6.1%) (Table 1).

**Table 1:** Pediatricians' demographics, workplace, their vaccine status and vaccine opinions.

Variable	Private (*n* = 299)	Workplace Public (*n* = 91)	Public and Private (*n* = 592)	Total (*n* = 982)
Own vaccine status [Incomplete], %	62.5	65.9	55.9	58.9
Medical residency experience [Yes], %	90.6	92.3	90.4	90.6
Master's degree [Yes], %	14.7	40.7	26.2	24.0
Doctorate degree [Yes], %	3.3	20.9	13.0	10.8
Gender [Female], %	74.2	80.2	78.5	77.4
Gender [Male], %	25.8	19.8	21.3	22.5
Gender [Non-binary], %	0.0	0.0	0.2	0.1
**Regarding SBP-recommended vaccines not available in the PNI**				
Recommend some to selected patients, %	9.0	47.3	15.7	16.6
Recommend some to all patients, %	9.7	11.0	16.0	13.6
Recommend all to all patients,%	80.6	37.4	67.7	68.8
Does not recommend any to any patient, %	0.7	4.4	0.5	0.9
**Vaccines are safe**				
Agree, %	14.7	22.0	16.7	16.6
Disagree, %	1.3	2.2	2.2	1.9
Neutral, %	4.3	3.3	5.6	5.0
Strong Agree, %	74.2	69.2	72.1	72.5
Strong Disagree, %	5.4	3.3	3.4	4.0
**Pediatric Covid-19 vaccines are essential**				
Agree, %	5.7	5.5	7.6	6.8
Disagree, %	3.7	5.5	4.7	4.5
Neutral, %	6.7	1.1	6.2	5.9
Strong Agree, %	80.3	81.3	77.0	78.4
Strong Disagree, %	3.7	6.6	4.4	4.4

### The factor structure of pediatrician vaccine hesitancy and misconceptions

Participants’ viewpoints on vaccines were assessed using an 11-statement Likert scale. To comprehensively explore pediatricians’ stances on vaccine-related issues, the authors divided the factor structure into two sections. The first section included five statements extracted from the Vaccine Hesitancy 5C scale. In the second section, six additional statements were used to explore beliefs related to misconceptions about vaccines.

#### Factorial structure of pediatrician responses to the 5C hesitancy scale

The authors evaluated the feasibility of the 5C vaccine hesitancy scale among pediatricians using a concise scale based on the findings by Betsch et al., which demonstrate the validity of the scale with just 5 items, one for each of the 5Cs.^10^ The items clustered into two factors instead of the expected five. Items related to complacency, collective responsibility, and constraints were grouped into one factor with factor loadings of 0.67, 0.41, and 0.35, respectively. The item about calculation formed another factor with a factor loading of 0.69. Lastly, the item concerning confidence exhibited factor loadings below the threshold of 0.3. Factor loading values below 0.5 indicate a poor correlation between items. This suggests that the scale's original structure may not hold in this sample. Foremost, the overall KMO value of 0.6 indicates that this factor structure is not well-suited for Factor Analysis. The RMSEA of 0.06, though slightly above the desired 0.05 threshold, shows a reasonable fit. However, the TLI value of 0.82 falls below the preferred 0.9 threshold, suggesting a suboptimal fit. While the model demonstrates the potential for enhancement, its slight deviations from targets indicate careful adjustments are worth considering to better capture the underlying variable relationships. Taking these results into account, the authors have arrived at the conclusion that the 5Cs scale has no positive construct validity evidence in the present sample. Due to these limitations, the authors refrained from conducting further analyses involving this factor.

#### Factorial structure of misconceptions about vaccines

Bartlett's Test of Sphericity yields a significant result (χ² = 1498, df = 15), confirming correlations among variables. The KMO value of 0.71 signifies adequate data suitability for factor analysis.

The factor structure converged into two dimensions. Factor 1, named “Misconceptions about COVID-19 Vaccines,” grouped items related to concerns about pediatric COVID-19 vaccines being experimental (loading of 0.81), potential DNA alteration by the mRNA COVID-19 vaccine (loading of 0.74), and the importance of children receiving the COVID-19 vaccine (loading of 0.58). Factor 2, labeled “Misconceptions towards other vaccines,” clustered items associated with misconceptions about different vaccines: belief in the MMR vaccine's link to autism (loading of 0.85), the idea of the Rotavirus vaccine causing milk protein allergy (loading of 0.66), and the perception that the HPV vaccine administered in adolescence may affect sexual life onset (loading of 0.52). The RMSEA indicated a value of 0.049 [CI 90% = 0.022 – 0.079], suggesting a reasonable fit between the proposed model and the data. The TLI score of 0.975 indicates a robust fit. The χ² test yielded a value of 4 with 0.009 degrees of freedom and a p-value of 0.009, signifying statistical significance. Overall, both factors demonstrated satisfactory internal consistency and reliability.

Next, the authors employed PCA to merge the items in same factors into two different scales. By transforming individual responses into a unified measure, PCA simplified the interpretation of these findings. The “Misconceptions towards other vaccines” scale was constructed ranging from -0.212 to 10.70 (M = 1.43, SD = 0.80), with a McDonald's ω coefficient of 0.75 indicating reasonable item consistency. The “Misconceptions about COVID-19 vaccines” scale was constructed ranging from -0.519 to 4.70 (M = 1.08; SD = 0.37), with a McDonald's ω coefficient of 0.73 also suggesting reasonable consistency.

### Relationship between pediatricians’ residency experience and vaccine status with misconceptions about COVID-19 vaccines

In the GLM analysis, the authors investigated how Pediatricians’ Residency Experience, Gender and Vaccine Status influence their beliefs and misconceptions about COVID-19 vaccines. The authors added a constant to the raw data of the dependent variable for conducting the GLM analysis, as the Gamma distribution only accepts non-zero positive values. The “Misconceptions about COVID-19 vaccines” score required this adjustment.^11^ Once the analyses were completed, the authors subtracted the constant from the coefficients to report the data accurately.^10^ The model's explanatory power is weak (Nagelkerke's R2 = 0.03). The model's intercept is at 10.16 (95% CI [9.94, 10.39], t(977) = 87.69, *p <* .001).

**Figure 1:**
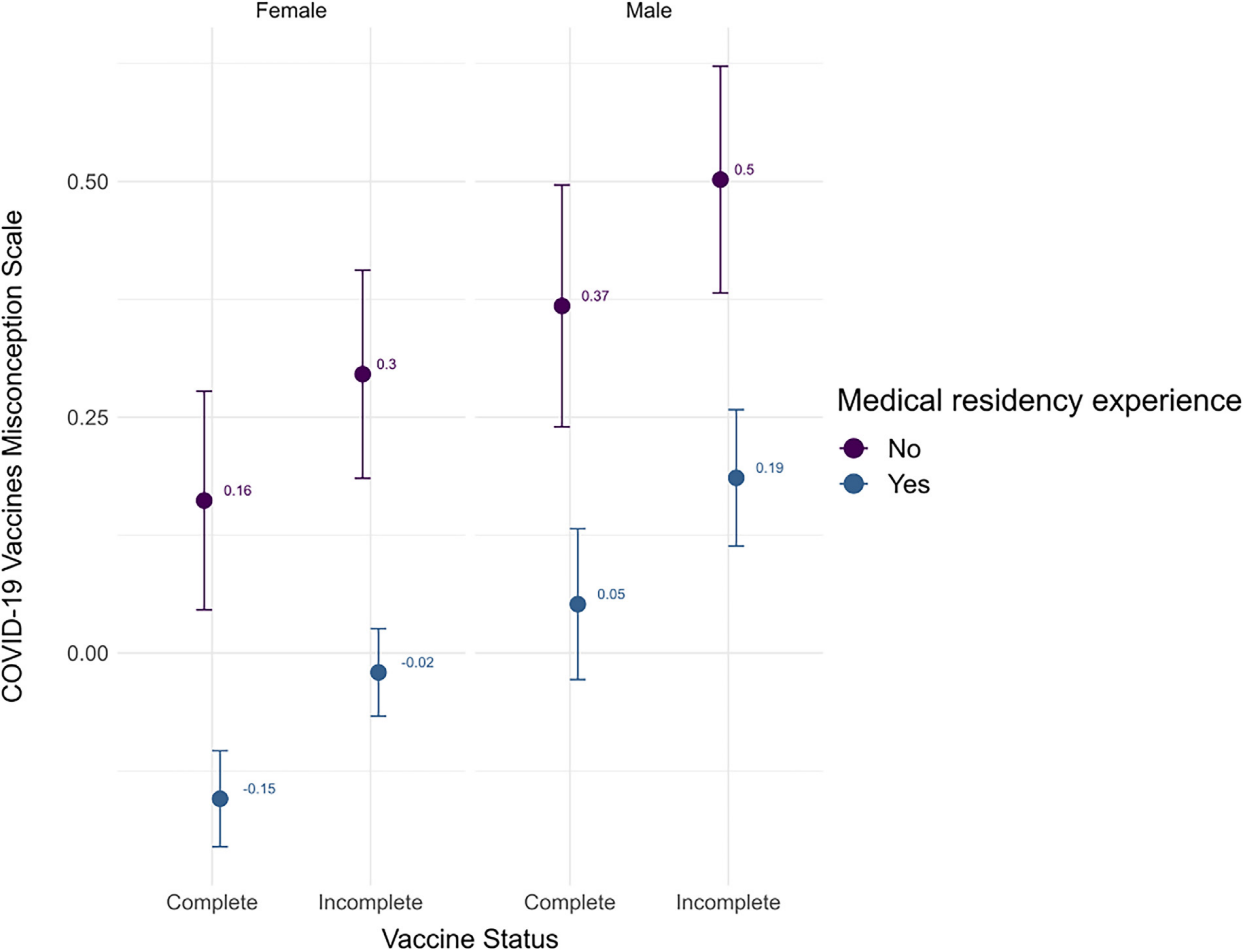
Impact of vaccine status and medical residency experience on misconceptions about COVID-19 vaccines scale.

Within this model (Figure 1):

1. The effect of Vaccine status [Incomplete] is statistically significant and positive (beta = 0.13, 95% CI [8.15e-03, 0.26], t(977) = 2.09, p = 0.036; Std. beta = 0.13, 95% CI [8.15e-03, 0.26]).
2. The effect of Gender [Male] is statistically significant and positive (beta = 0.21, 95% CI [0.06, 0.36], t(977) = 2.70, p = 0.007; Std. beta = 0.21, 95% CI [0.06, 0.36]).
3. The effect of Medical residency experience [Yes] is statistically significant and negative (beta = -0.32, 95% CI [-0.54, -0.10], t(977) = -2.84, p = 0.004; Std. beta = -0.32, 95% CI [-0.54, -0.10]).

Standardized parameters were obtained by fitting the model on a standardized version of the dataset. 95% Confidence. Intervals (CIs) and p-values were computed using a Wald t-distribution approximation.

### Analysis of misconceptions towards other vaccines

The “Misconceptions Towards Other Vaccines” scale did not yield statistically significant results with any of the independent variables collected in the study.

### Associations among pediatrician profiles

**Figure 2:**
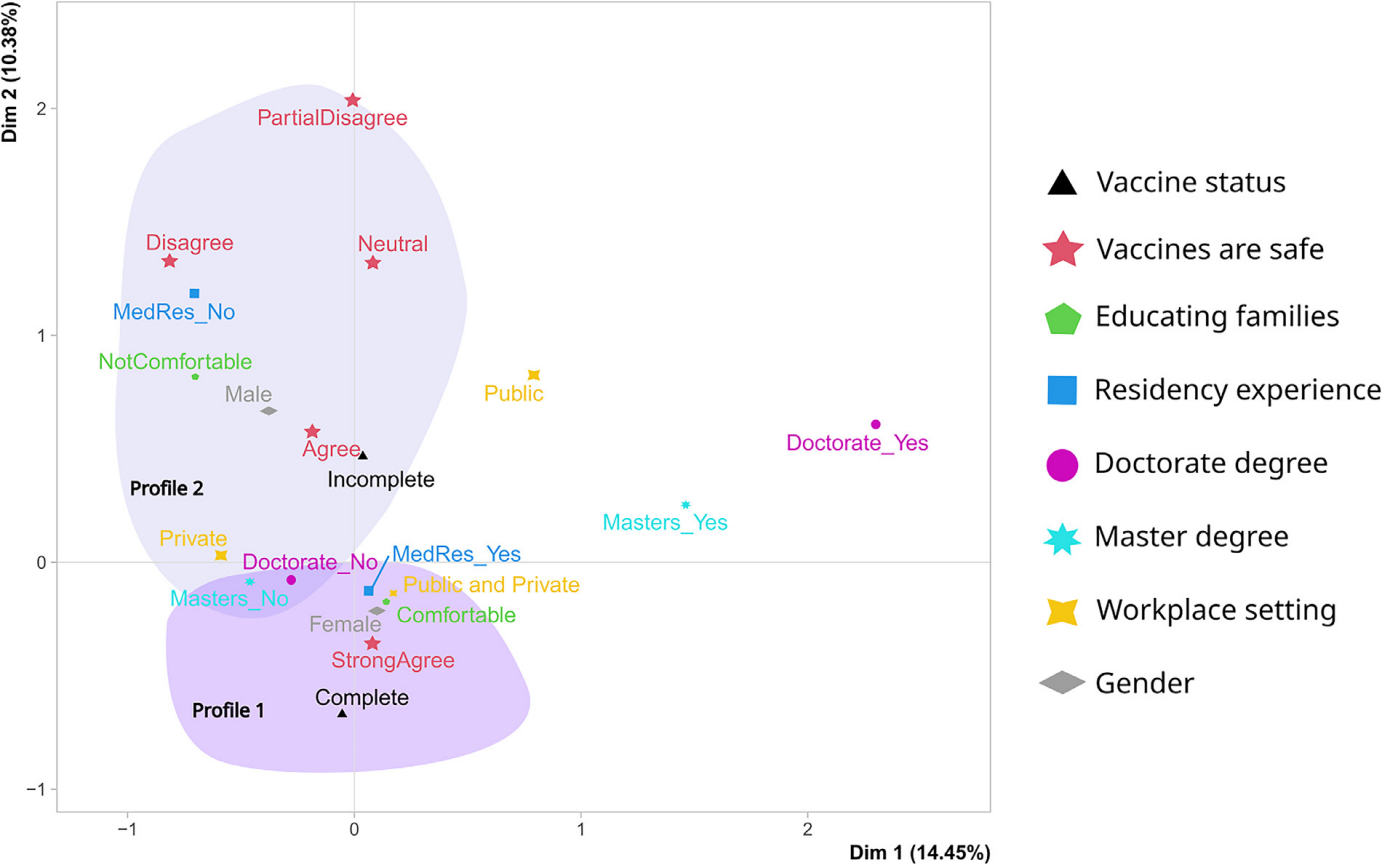
**Multiple Correspondence Analysis of Pediatricians' Profiles:** The biplot represents the profiles of pediatricians based on their responses to various factors: their own vaccination status (triangle), perception of vaccine safety (star), comfort level in educating families (pentagon), medical residency experience (square), possession of a doctorate (purple dot) or master's degree (cyan asterisk), workplace setting (star shape: public, private, or both), and gender (diamond). The two dimensions explain 14.45% and 10.38% of the variability, respectively.

In assessing group differences, the authors employed correspondence analysis (Figure 2). This approach allowed us to explore associations among the following categorical variables: (a) vaccine status; (b) vaccines are safe; (c) comfort in educating families; (d) medical residency experience; (e) academic degrees; (f) workplace setting; and (g) Gender.

The correspondence analysis revealed two distinct profiles among pediatricians. Profile 1 represents female pediatricians with medical residency but without a master's or doctorate degree. They are confident in educating families about vaccines, work across public and private healthcare sectors, have complete personal vaccination status, and trust in vaccine safety.

Profile 2 depicts male pediatricians without medical residency who face challenges in vaccine education, with incomplete personal vaccination status. They predominantly work in the private sector, lack advanced degrees, and have differing views on vaccine safety. These findings highlight varying attitudes and practices within the pediatrician community regarding vaccine education, experience, and safety perceptions.

The first two dimensions of the analysis account for 24.83% of the total dataset's variability, meaning that this percentage of the overall variability is explained by the plane. Although a small proportion, it is higher than the reference value of 19.67%, suggesting significant variability explained by this plane. This reference value is based on the 0.95-quantile of the inertia percentages distribution obtained by simulating 5735 data tables of equivalent size on the basis of a uniform distribution. The graph analysis does not identify any outliers.

## Discussion

Brazil's PNI has improved public health by helping to eliminate and control diseases and increase life expectancy. Globally, vaccines save lives and offer economic benefits.^12^, ^13^, ^14^, ^15^ However, their success relies on public acceptance and adherence to vaccination programs.^16^^,^^17^

Research by Mohanty et al. highlighted these challenges and proposed strategies such as community-based interventions to address vaccine hesitancy.^4^ The present study found that fully vaccinated pediatric primary care providers (PCPs) with residency experience felt more confident in handling vaccine-hesitant families, suggesting that vaccination status and residency experience enhance competence in managing hesitancy. They also felt more comfortable employing improved communication skills and strategies to address vaccine hesitancy. This suggests that trust in vaccine, vaccine status and residency experience may enhance PCPs’ competence and credibility in managing vaccine hesitancy. The present findings are consistent with the discovery made by Mondal P and Sinharoy A, indicating that pediatricians’ affirmative recommendation was the most influential predictor of caregivers’ vaccine acceptance, followed by self-COVID-19 vaccination status and post-vaccination side effects.^18^

Moreover, other studies emphasized the pivotal role of pediatric healthcare providers in tackling vaccine hesitancy. The research pointed out that healthcare providers can play a crucial role by focusing on parental education and improving communication. By better educating parents about the safety and efficacy of vaccines, and by enhancing their communication skills, healthcare providers can help alleviate vaccine hesitancy within the pediatric population.^19^^,^^20^

Suryadevara's 2015 research delved into the revelation that vaccine hesitancy also exists among pediatric healthcare providers themselves. This hesitancy is driven by concerns related to vaccine safety, efficacy, and misconceptions about vaccines, such as the belief that vaccines can lead to autism or DNA modification. The study emphasized that addressing this hesitancy among healthcare providers is essential, as it can have a direct impact on the recommendations and attitudes they convey to parents.^21^

The present study provides a valuable perspective addressing misconceptions about vaccines, especially COVID-19 vaccines, among Brazilian pediatricians. These results suggest that pediatricians with an incomplete vaccination status score, on average, 0.15 points higher on the scale measuring misconceptions about COVID-19 vaccines when compared to their colleagues with a complete vaccination status. These findings indicate that pediatricians who keep their vaccinations up to date may exhibit lower levels of hesitancy, which aligns with the concept of healthcare professionals serving as vaccine advocates. These results suggest that pediatricians’ trust in vaccines may affect their vaccination status and recommendations regarding vaccines.^8^^,^^22^, ^23^, ^24^, ^25^ There are relevant psychological and social mechanisms that explain such relations, such as the motivation to balance cognition and behavior as explained by cognitive balance and cognitive dissonance.^26^

In the same sense, pediatricians with medical residency experience score, on average, 0.33 points lower on the scale measuring misconceptions about COVID-19 vaccines when compared to their colleagues without medical residency experience. This suggests that pediatricians without medical residency experience exhibit higher levels of hesitancy. Although this finding is intriguing, it warrants further exploration and contextualization. Research has previously highlighted that medical education, which often includes residency, can significantly influence a physician's attitudes toward vaccines.^27^, ^28^, ^29^ Hence, this result may be attributed to the impact of formal medical training.

The two profiles identified in the multiple correspondence analysis align with the results obtained from the linear models and corroborate findings in the existing literature.^8^^,^^30^ These findings underscore the presence of diverse attitudes and practices within the pediatrician community concerning vaccine education, professional experience, and perceptions of vaccine safety.

The present study has two main limitations. First, the non-normal distribution of residuals in the GLM model may introduce bias, affecting the reliability and generalizability of these results. To address this, the authors used a model with a Gamma distribution. Second, the use of convenience sampling through email and WhatsApp recruitment could lead to selection bias, potentially compromising the representativeness of this sample.

Despite these limitations, the present study highlights a significant correlation between vaccination status and medical residency experience among pediatricians, which influences their perceptions of COVID-19 vaccines. Fully vaccinated pediatricians, especially those with residency experience, tend to have fewer misconceptions, demonstrating the positive impact of formal medical training on vaccine understanding. In contrast, pediatricians with incomplete vaccination status and no residency experience exhibit higher levels of misconceptions. These findings emphasize the importance of comprehensive vaccination strategies tailored to healthcare providers' backgrounds to enhance vaccine confidence effectively.

Future studies should explore additional factors influencing vaccine hesitancy among health professionals and across different specialties. It is also crucial to develop appropriate psychometric measures that are culturally and socially relevant to evaluate vaccine hesitancy among health professionals and the general Brazilian population. These efforts are essential for creating effective strategies to mitigate vaccine hesitancy in the current context.

## Funding

This work was supported by the Brazilian Society of Pediatrics.

## Conflicts of interest

The authors declare that Luiz Gustavo de Almeida, Ronaldo Pilati and Eduardo Jorge da Fonseca Lima have no competing interests to disclose. Natalia Pasternak Taschner participated in a board for innovation and intellectual property at Janssen Brazil, from November 2022-23. Her compensation was donated in full to a Brazilian charity. Renato Kfouri took part in the advisory board of Pfizer and worked as a Sub-investigator for the AstraZeneca-Oxford Vaccine trial in the Brazilian setting.
